# Supplementary material for: Distributional bias compromises leave-one-out cross-validation
Source: Sci Adv. 2025 Nov 28;11(48):eadx6976. doi: 10.1126/sciadv.adx6976 (PMC12662204; doi:10.1126/sciadv.adx6976)
Supplement: Supplementary file 1 — Notes S1 to S3 Figs. S1 to S8 References [file sciadv.adx6976_sm.pdf]

Science Advances
AAAS

Supplementary Materials for

Distributional bias compromises leave-one-out cross-validation

George I. Austin *et al.*

Corresponding author: Tal Korem, tal.korem@columbia.edu

Sci. Adv. **11**, eadx6976 (2025)
DOI: 10.1126/sciadv.adx6976

This PDF file includes:

Notes S1 to S3
Figs. S1 to S8
References

Supplementary Text

Supplementary Note 1 | RLOOCV resolves distributional bias

Proposition 1. *Let* $\mathscr{D} = \{(x_i, y_i)\}_{i=1}^n$ *be a training dataset, and* $\tau = \{(x_j, y_j)\}_{j=1}^m$ *be a test dataset, with* (x_i, y_i) *sampled from some joint probability* $P_{X\mathbf{Y}}$*. Then, for a fair evaluation of the generalization performance of a model trained using only the training data,* $\mathscr{D}$ *must be statistically independent from* τ.

If all samples are i.i.d., then this condition is already met. Some scenarios may compromise this assumption, such as the analysis of a collection of samples drawn from different batches in which the underlying biases compromise the i.i.d. assumption *(8, 48)*. This proposition ensures that the estimated performance is not biased by prior exposure to test labels, and violating this independence makes the evaluation unrepresentative of scenarios in which true labels are not known. We note that this condition is met in standard out-of-sample evaluations.

Proposition 2. *Consider a dataset* $\mathscr{D} = \{(x_i, y_i)\}_{i=1}^n$*, where each* (x_i, y_i) *are i.i.d. from some join probability* $P_{X\mathbf{Y}}$*. Consequently, each* x_i *follows some conditional probability* $P_{X|\mathbf{Y}}$*. During any cross-validation scheme, validation on a held-out sample with a label* y_i *should be independent of the training dataset used.*

This is an extension of Proposition 1, applied to leave-one-out cross-validation (LOOCV).

Theorem 1. *Evaluations obtained via LOOCV are not representative of out-of-sample generalization, because any training dataset in LOOCV is not independent of the held-out sample it is validated on.*

Proof. Consider once again a dataset $\mathscr{D} = \{(x_i, y_i)\}_{i=1}^n$, where each (x_i, y_i) are drawn i.i.d. from some join probability $P_{X\mathbf{Y}}$, and each x_i follows some conditional probability $P_{X|\mathbf{Y}}$. We define the list of labels, $\mathbf{Y} = \{y_i\}_{i=1}^n$, and the set of all unique labels $\mathscr{Y}$, such that each $y_i \in \mathscr{Y}$. The conditional probability of the dataset $\mathscr{D}$ given $\mathbf{Y}$ is:

$$\Pr(\mathscr{D}|\mathbf{Y}) = \prod_{i=1}^{n} \Pr(x_i|y_i)$$

Additionally, denote $\mathscr{D}^{-i} = \{(x_j, y_j)\}_{j \neq i}^n$ as the dataset $\mathscr{D}$ without sample i, which is, under LOOCV, the training dataset used before testing on the sample i. Denote $\Pr(y_i)$ as the probability of observing a particular test instance y_i, and $\Pr(\mathscr{D}^{-i})$ as the probability of observing a particular training set $\mathscr{D}^{-i}$.

For any dataset $\mathscr{D}$ and $a \in \mathscr{Y}$, consider the LOOCV fold with a test of either $y_i = a$ or $y_i \neq a$, and assuming all x_i are i.i.d.:

$$\Pr(\mathscr{D}^{-i}|\mathbf{Y}, y_i = a) = \prod_{j \neq i}^{n} \Pr(x_j|y_j) = \frac{\prod_{j=1}^{n} \Pr(x_j|y_j)}{\Pr(x_i|y_i = a)}$$

$$\Pr(\mathscr{D}^{-i}|\mathbf{Y}, y_i \neq a) = \frac{\prod_{j=1}^{n} \Pr(x_j|y_j)}{\Pr(x_i|y_i \neq a)}$$

Therefore:

$$\Pr(\mathscr{D}^{-i}|\mathbf{Y}, y_i = a) \cdot \Pr(x_i|y_i = a) = \Pr(\mathscr{D}^{-i}|\mathbf{Y}, y_i \neq a) \cdot \Pr(x_i|y_i \neq a)$$

Assuming that, under the joint probability $P_{X\mathbf{Y}}$, x_i and y_i are not independent, then $\Pr(x_i|y_i = a) \neq \Pr(x_i|y_i \neq a)$. This then implies that:

$$\Pr(\mathscr{D}^{-i}|\mathbf{Y}, y_i = a) \neq \Pr(\mathscr{D}^{-i}|\mathbf{Y}, y_i \neq a)$$

Since, via marginalization, $\Pr(\mathscr{D}^{-i}|\mathbf{Y}) = \sum_{b\in\mathscr{Y}} \Pr(y_i = b|\mathbf{Y}) \Pr(\mathscr{D}^{-i}|\mathbf{Y}, y_i = b)$:

$$\Pr(\mathscr{D}^{-i}|\mathbf{Y}) = \Pr(y_i = a|\mathbf{Y}) \Pr(\mathscr{D}^{-i}|\mathbf{Y}, y_i = a) + \Pr(y_i \neq a|\mathbf{Y}) \Pr(\mathscr{D}^{-i}|\mathbf{Y}, y_i \neq a)$$

Substituting in the inequality, assuming that the probability spaces are not trivial or constant, which is implied based on some assumed relationship between the X_i and y_i:

$$\Pr(\mathscr{D}^{-i}|\mathbf{Y}) \neq \Pr(y_i = a|\mathbf{Y}) \Pr(\mathscr{D}^{-i}|\mathbf{Y}, y_i = a) + \Pr(y_i \neq a|\mathbf{Y}) \Pr(\mathscr{D}^{-i}|\mathbf{Y}, y_i = a)$$
$$\Pr(\mathscr{D}^{-i}|\mathbf{Y}) \neq \Pr(\mathscr{D}^{-i}|\mathbf{Y}, y_i = a) \cdot \left(\Pr(y_i = a|\mathbf{Y}) + \Pr(y_i \neq a|\mathbf{Y}) \right)$$

Therefore, since $\Pr(y_i = a|\mathbf{Y}) + \Pr(y_i \neq a|\mathbf{Y}) = 1$:

$$\Pr(\mathscr{D}^{-i}|\mathbf{Y}) \neq \Pr(\mathscr{D}^{-i}|\mathbf{Y}, y_i = a)$$
$$\Pr(\mathscr{D}^{-i}|\mathbf{Y}) \cdot \Pr(y_i|\mathbf{Y}) \neq \Pr(\mathscr{D}^{-i}|\mathbf{Y}, y_i = a) \cdot \Pr(y_i|\mathbf{Y})$$
$$\Pr(\mathscr{D}^{-i}|\mathbf{Y}) \cdot \Pr(y_i|\mathbf{Y}) \neq \Pr(\mathscr{D}^{-i} \cap y_i|\mathbf{Y})$$

Therefore, given a dataset for which LOOCV is being performed, $\mathscr{D}^{-i}$ and y_i are not independent.

This dependence can also be observed by simply tracking the total number of labels available during training at each iteration. For any dataset $\mathscr{D}$ and $a \in \mathscr{Y}$, consider the total number of samples with $y_i = a$ denoted as $|\{(x_i, y_i) \in \mathscr{D}|y_i = a\}|$. Thus, for an LOOCV fold in which the test set is $y_j \neq a$:

$$|\{(x_k, y_k) \in \mathscr{D}^{-j}|y_k = a\}| = |\{(x_i, y_i) \in \mathscr{D}|y_i = a\}|$$

And for another LOOCV fold in which the test set is $y_i = a$:

$$|\{(x_k, y_k) \in \mathscr{D}^{-i}|y_k = a\}| = |\{(x_i, y_i) \in \mathscr{D}|y_k = a\}| - 1$$

Therefore:

$$|\{(x_k, y_k) \in \mathscr{D}^{-i}|y_k = a\}| \neq |\{(x_k, y_k) \in \mathscr{D}^{-j}|y_k = a\}|$$

which implies that the possible events for $\mathscr{D}^{-i}$ and $\mathscr{D}^{-j}$ are disjoint. This indicates that for some D^{-i}, if $Pr(D^{-i}|y_k = a) > 0$, then $Pr(D^{-i}|y_k \neq a) = 0$. Thus, $Pr(D^{-i}|y_k = a) \neq Pr(D^{-i}|y_k \neq a)$, and following the same construction as before, this implies that $\Pr(\mathscr{D}^{-i}|\mathbf{Y}) \cdot \Pr(y_i|\mathbf{Y}) \neq \Pr(\mathscr{D}^{-i} \cap y_i|\mathbf{Y})$. Therefore, this once again proves that $\mathscr{D}^{-i}$ and y_i are not independent.

□

We note that while we prove a lack of independence, it is possible for this non-independence to bias a machine learning model to produce both over-optimistic (such as in **Fig. 1**), or over-pessimistic results (such as in **Figs. 2-5**). Although most practical machine learning models will produce a pessimistic bias, providing concrete bounds on this bias requires an assumption of a specific model structure.

Theorem 2. *Evaluations obtained via rebalanced leave-one-out cross-validation (RLOOCV) preserve independence between training sets and held-out test labels.*

Proof. We follow the same construction as in Theorem 1, with a dataset $\mathscr{D} = \{(x_i, y_i)\}_{i=1}^n$, where (x_i, y_i) are i.i.d. from some join probability $P_{X\mathbf{Y}}$, each x_i following some conditional probability $P_{X|\mathbf{Y}}$. Given a dataset with a specified set of $\mathbf{Y}$ labels, where each $y_i = a$ (for some $a \in \mathscr{Y}$), the conditional probability of observing a dataset $\mathscr{D}$ is:

$$\Pr(\mathscr{D}|\mathbf{Y}) = \prod_{i=1}^{n} \Pr(x_i|y_i)$$

In RLOOCV for binary classification (i.e., $\mathscr{Y} = \{0,1\}$), for any test set $y_i = 0$, an additional sample k is removed from the training set where $y_k = 1$ (and $y_k = 0$ when $y_i = 1$). Denote $\mathscr{D}^{-(i,k)} = \{(x_j, y_j)\}_{j \notin (i,k)}^n$ as the dataset $\mathscr{D}$ without samples i and k. Then all possible training datasets are represented by $\mathscr{D}^{-(i,j)}$, for some $y_i = 0$ and $y_j = 1$, with one of the indices held out for testing and the other held out for rebalancing. We note that similar constructions apply for multiclass classification.

Therefore, for any dataset $\mathscr{D}$, consider the RLOOCV training folds with a test set of either $y_i = 0$ or $y_i = 1$, and assuming all x_i are i.i.d.:

$$\Pr(\mathscr{D}^{-(i,k)}|\mathbf{Y}, y_i = 0) = \prod_{j \neq i,k}^{n} \Pr(x_j|y_j) = \frac{\prod_{j=1}^{n} \Pr(x_j|y_j)}{\Pr(x_i|y_i = 0)\Pr(x_k|y_k = 1)}$$

$$\Pr(\mathscr{D}^{-(i,k)}|\mathbf{Y}, y_i = 1) = \frac{\prod_{j=1}^{n} \Pr(x_j|y_j)}{\Pr(x_i|y_i = 1)\Pr(x_k|y_k = 0)}$$

Therefore, once again solving for and substituting the $\prod_{j=1}^{n} \Pr(x_j|y_j)$:

$$\Pr(\mathscr{D}^{-(i,k)}|\mathbf{Y}, y_i = 0)\Pr(x_i|y_i = 0)\Pr(x_k|y_k = 1) = \Pr(\mathscr{D}^{-(i,k)}|\mathbf{Y}, y_i = 1)\Pr(x_i|y_i = 1)\Pr(x_k|y_k = 0)$$

After canceling out the $\Pr(x_i|y_i = 1)\Pr(x_k|y_k = 0)$ terms:

$$\Pr(\mathscr{D}^{-(i,k)}|\mathbf{Y}, y_i = 0) = \Pr(\mathscr{D}^{-(i,k)}|\mathbf{Y}, y_i = 1)$$

Since we can marginalize across $\Pr(\mathscr{D}^{(-i,k)})$, substitute in the above equality for some $a \in \{0,1\}$, and factoring:

$$\Pr(\mathscr{D}^{-(i,k)}|\mathbf{Y}) = \sum_{y \in \{0,1\}} \Pr(y_i)\Pr(\mathscr{D}^{-(i,k)}|\mathbf{Y}, y_i)$$

$$\Pr(\mathscr{D}^{-(i,k)}|\mathbf{Y}) = \Pr(\mathscr{D}^{-(i,k)}|\mathbf{Y}, y_i = a)\left[\sum_{y \in \{0,1\}} \Pr(y_i)\right]$$

Since $\left[\sum_{y \in \{0,1\}} \Pr(y_i)\right] = 1$:

$$\Pr(\mathscr{D}^{-(i,k)}|\mathbf{Y}) = \Pr(\mathscr{D}^{-(i,k)}|\mathbf{Y}, y_i)$$

$$\Pr(\mathscr{D}^{-(i,k)}|\mathbf{Y})\Pr(y_i|\mathbf{Y}) = \Pr(\mathscr{D}^{-(i,k)}|\mathbf{Y}, y_i = a)\Pr(y_i|\mathbf{Y})$$

$$\Pr(\mathscr{D}^{-(i,k)}|\mathbf{Y})\Pr(y_i|\mathbf{Y}) = \Pr(\mathscr{D}^{-(i,k)} \cap y_i|\mathbf{Y})$$

Therefore, by the definition of independence, training on a dataset $\mathscr{D}^{(-i,k)}$ is independent of the held-out label y_i in

RLOOCV.

We additionally note that the total amount of labels from each class is also constant across all training iterations in RLOOCV, as every training dataset uses exactly one fewer positive lable than the total amount in the dataset $\mathscr{D}$. $\square$

Corollary 1. *The rebalancing of RLOOCV cannot introduce an overly optimistic or pessimistic bias between the trained models and the held-out validation labels.*

Since $D^{-(i,k)}$ and y_i are proven to be statistically independent in Theorem 2, for any $i, j, \mathbf{Y}, \mathscr{D}$, then $\Pr(\mathscr{D}^{-(i,k)}|\mathbf{Y}) = \Pr(\mathscr{D}^{-(j,k)}|\mathbf{Y})$. Therefore:

$$\mathbb{E}[\mathscr{D}^{-(i,k)}|\mathbf{Y}] = \mathbb{E}[\mathscr{D}^{-(j,k)}|\mathbf{Y}]$$

Thus, RLOOCV cannot introduce any new bias in training datasets during the correction for distributional bias, as there expectation of the training datasets across folds are identical, regardless of the held-out labels. Therefore, RLOOCV is unbiased.

Supplementary Note 2 | Generalizing RLOOCV to RLPOCV and RKFold

Consider a dataset consisting of T positive samples, F negative samples, and a desired P left-out schema. The simple approach to rebalance LPOCV per held-out set is to randomly remove P cases from the training set with labels opposite those of the held-out set, such that every model is trained using exactly $T+F-2P$ observations, consisting of $T-P$ positive and $F-P$ negative samples. However, acknowledging that LPOCV would be considered in scenarios that are already data-sparse, we show that using stratification for the held-out test set, it is possible to develop a stratified RKFold schema that removes fewer samples from the training set, which can be especially important in a highly imbalanced dataset with few observations from one class. We consider the following:

- $N = T + F//P$ the total number of P-sized held-out groups.
- $T_C = T//N$, the total number of positive class observations that are guaranteed to be present in every left-out P group
- $F_C = F//N$, the total number of negative class observations that are guaranteed to be present in * every left-out P group.

Therefore, every held-out group consists of:

1. T_C positive samples.
2. F_C negative samples
3. $P-(T_C+F_C)$ remaining samples, which will cause label averages to vary across training folds, leading to distributional bias

Because components (1) and (2) are constant across all training folds, there is no need to subsample training datasets to match those held out samples. The only subsampling needed to ensure consistent class balances across all training folds is to remove samples opposite each case in (3). Therefore, when performing stratified RLPOCV, each training fold should use exactly:

- $T-P+F_C$ positive samples.
- $F-P+T_C$ negative samples.

To demonstrate a few examples, under this RLPOCV implementation, for a dataset with $T = 50, F = 50$, and $P = 5$, each model will be trained using exactly 47 T and 47 F samples. For a different dataset with $T = 10, F = 1000$, and $P = 2$, each model will be trained using exactly 9 T and 998 F samples. We note that in the case of $P = 1$, RLPOCV is identical to the RLOOCV implementation.

Supplementary Note 3 — Post-hoc standardization addresses distributional bias but may lead to performance inflation

An intuitive approach for correcting distributional bias could be to normalize (scale, z-score, etc.) a model's predictions on the held-out test set with respect to the same model's predictions on its training set. We describe this approach here as a cautionary tale, as it is unsuitable in certain settings. In some of our simulations, such as those analyzed with logistic regression, this approach performs exactly as intended, resulting in auROCs close to the expected 0.5 ($p = 0.14$ via one-sample t-test; **Fig. S8A**). However, in cases in which a model does not have the flexibility to precisely predict a class balance, for instance only being capable of predicting 0 or 1, then any normalization with respect to training label averages will create perfect rankings within all samples initially predicted at the same value. The simplest example of this is the K-Nearest Neighbor model with $K = 1$: When running this predictor through our same simulation framework but including a post-hoc z-score standardization to the training predictions, we observe auROCs larger than the intended 0.5 in every case ($p < 0.001$ via one- sample t-test; **Fig. S8B**), and with auROCs greater than 0.8 at extreme class imbalances. Although there are cases in which this normalization strategy is not problematic, we see no generalizable and concrete methods to ensure that elements of the more problematic models are not present in a given situation. Therefore, we conclude that there is always some risk of over-evaluation of machine learning performance when using a post-hoc normalization, and we recommend to avoid using it as a solution to distributional bias. Instead, RLOOCV solves this challenge without introducing any risk of overinflating results.

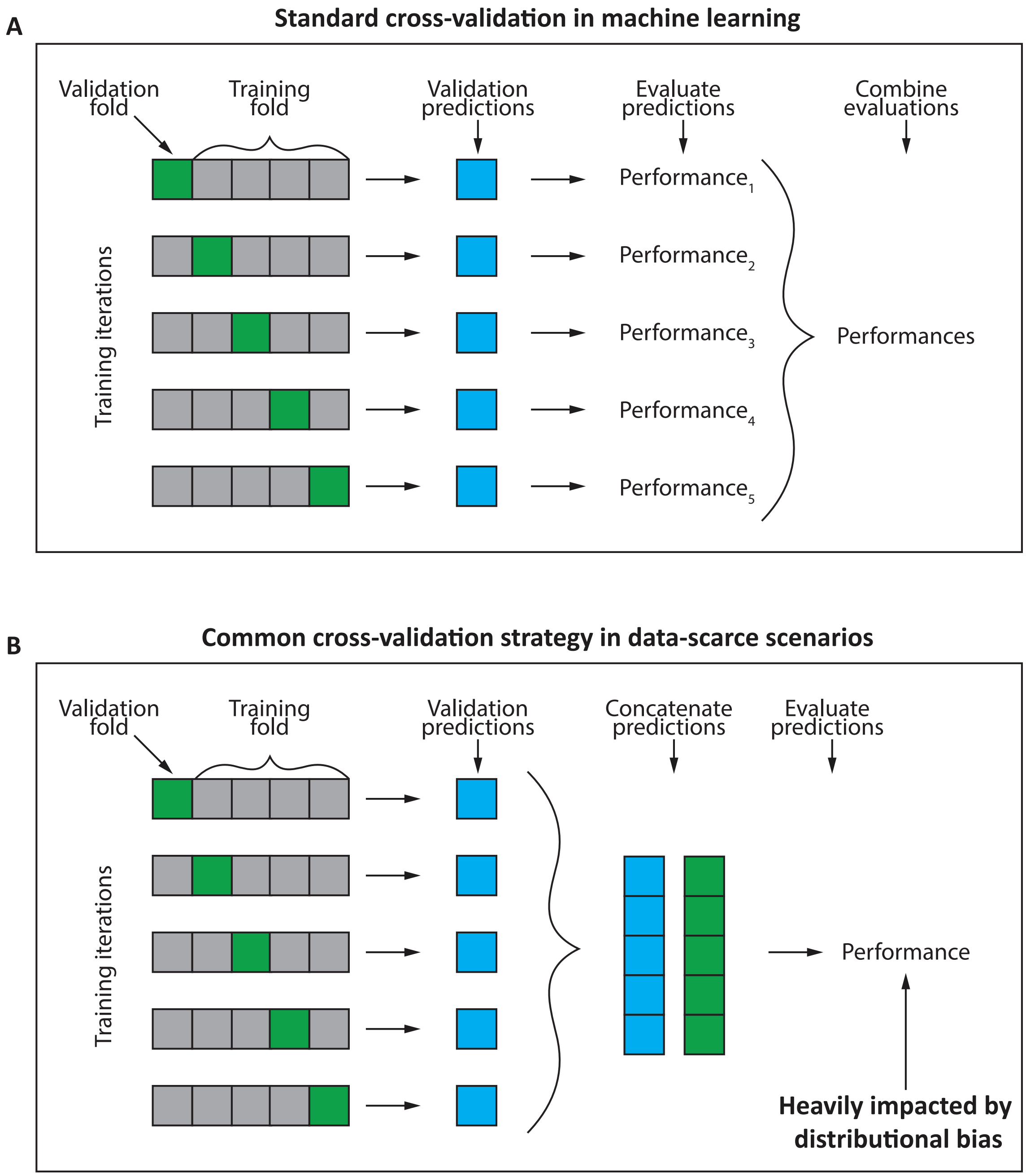

Fig. S1.

Comparison of cross-validation strategies. (**A**) Description of standard cross-validation approaches in machine learning, in which performance evaluation is performed separately for each held-out test set. (**B**) A commonly used cross-validation strategy in data-scarce scenarios, in which the evaluation across all folds is performed once on the concatenation of all predictions. The evaluation approach in (**B**) can be heavily impacted by distributional bias. Figure inspired by ref. (*6*).

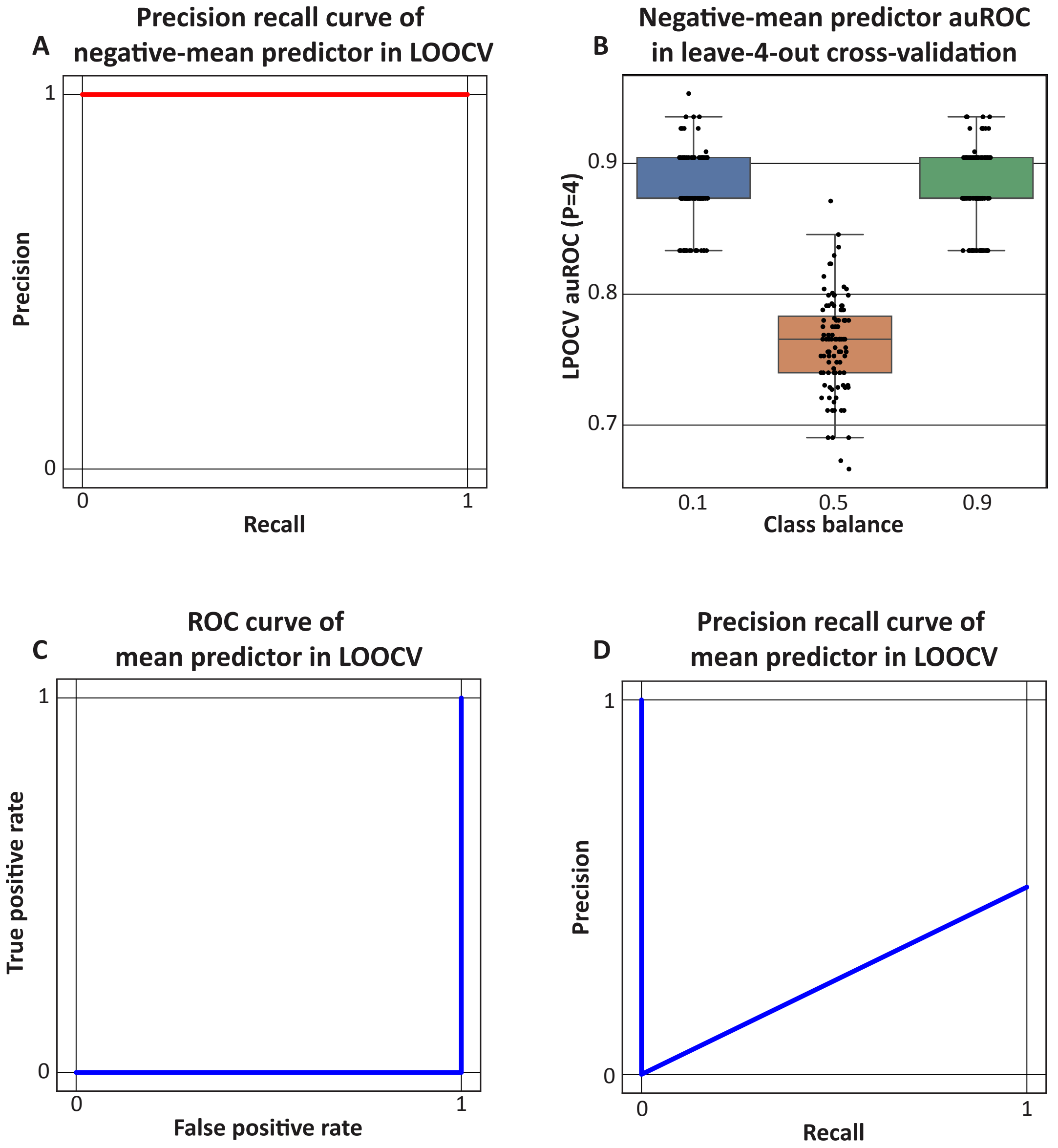

Fig. S2.

Impact of distributional bias across dummy models and evaluation metrics. (**A**) Results from the same model as in **Fig. 1B**, shown in a precision-recall curve, with an area under the curve of 1. (**B**) Box and swarm plot demonstrating results from the same set of models as in **Fig. 1C** for P=4 and class balances of 10%, 50%, and 90%. Box, IQR; line, median; whiskers, nearest point to 1.5*IQR. (**C,D**) ROC and PR curves similar to **Fig. 1B** and **Fig. S2A**, but for a predictor that outputs the label mean of the training set. Under LOOCV evaluation, for a class balance of 0.5, auROC=0 and auPR=0.25.

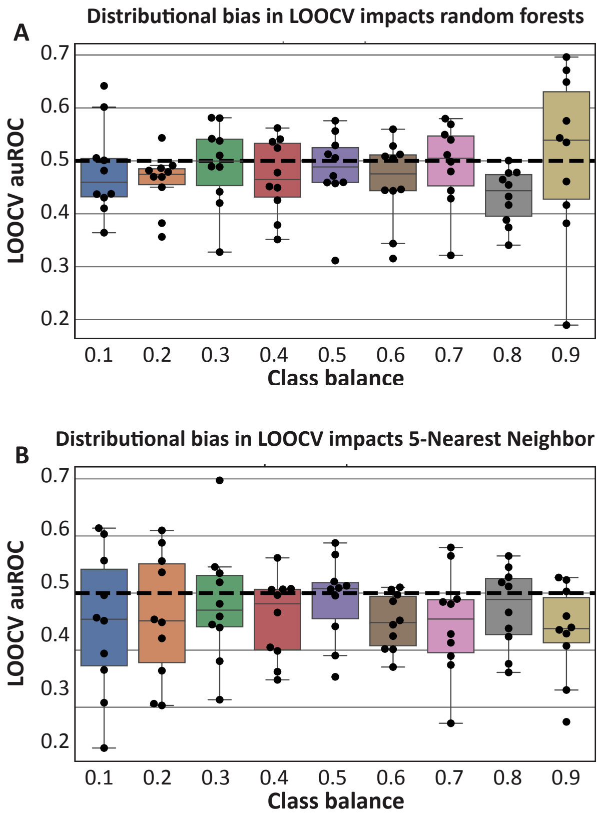

Fig. S3.

Distributional bias impacts random forest and 5-Nearest Neighbor models. (**A,B**) Boxplots demonstrating similar analyses as in **Fig. 2A**, but for random forest models (**A**), and K-Nearest Neighbors with K=5 (**B**). The resulting auROCs are consistently lower than 0.5 (p=0.009 via one-sample t-test vs. 0.5 for random forest, p=2.9×10^{-5} for K-Nearest Neighbors). Box, IQR; line, median; whiskers, nearest point to 1.5*IQR.

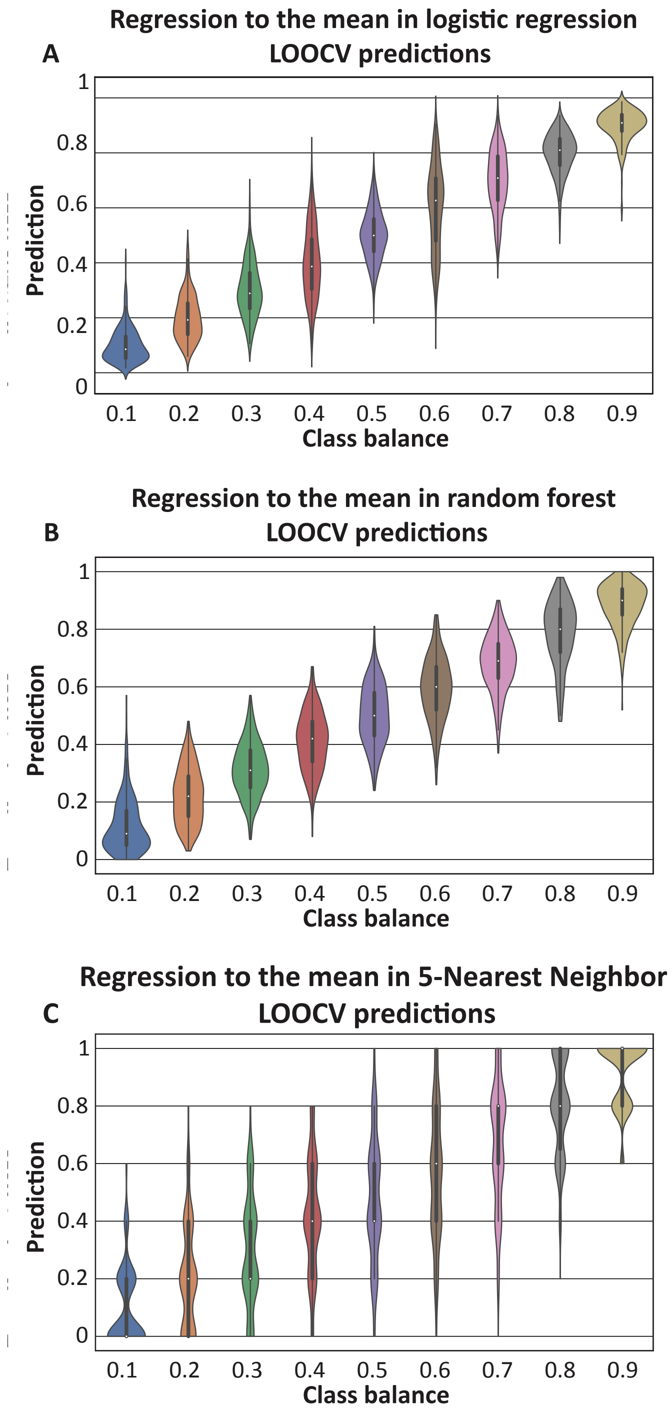

Fig. S4.

Regression to the mean in common machine learning models. (**A-C**) Violin plots showing predictions of logistic regression (**A**), random forest (**B**), and 5-Nearest Neighbors (**C**) models on randomly generated datasets in LOOCV, across different underlying class balances. All models used default scikit-learn parameters.

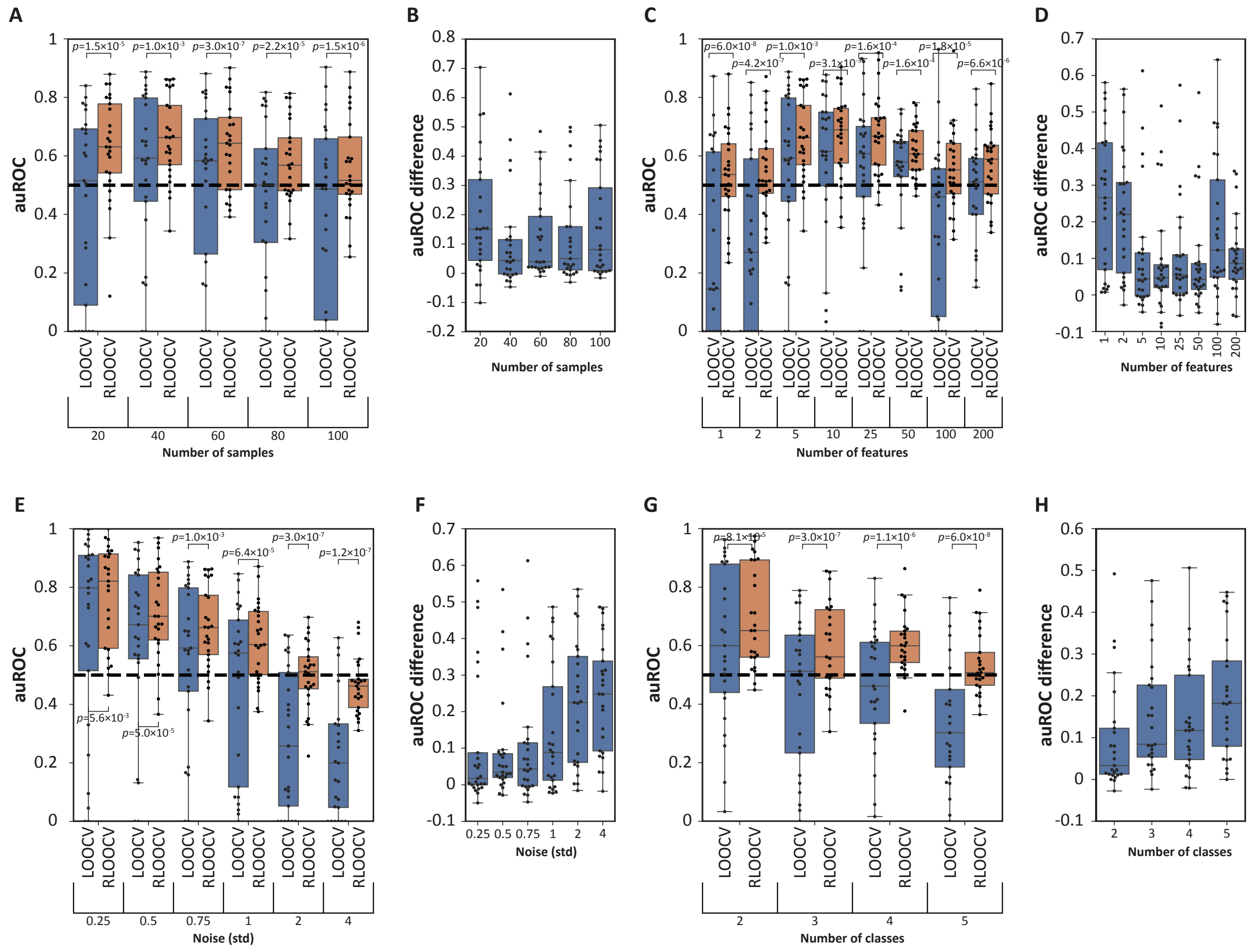

Fig. S5.

RLOOCV improves over LOOCV evaluated performances. Box and swarm plots of the auROCs of models evaluated with LOOCV and RLOOCV on simulated datasets with true associations with the labels (**Methods**), under varying number of samples (**A,B**), features (**C,D**), signal strength (**E,F**), and number of classes (**G,H**). Panels **A,C,E** and **G** show the auROCs of LOOCV and RLOOCV; panels **B,D,F** and **H** show the pairwise gain in auROC obtained using RLOOCV compared to LOOCV. All results are from a nested 5-fold tuning for L^2 logistic regression. Unless otherwise specified, the simulations used 40 samples, 5 features, noise (std) of 0.75, and 2 classes. Box, IQR; line, median; whiskers, nearest point to 1.5*IQR. p, two-sided Wilcoxon signed rank test comparing RLOOCV with LOOCV.

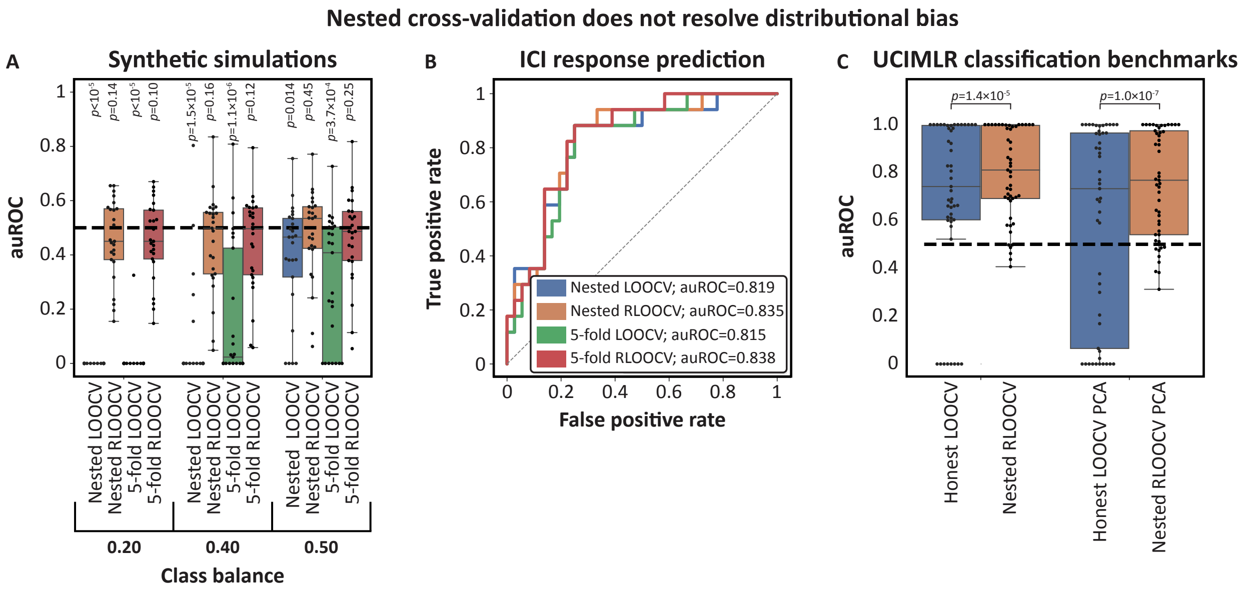

Fig. S6.

Distributional bias compromises nested cross-validation and Honest Leave-one-out. All analyses pertain to comparison of hyperparameter optimization schemes using nested cross-validation, including either: (1) outer LOOCV and inner LOOCV (known as "Honest LOOCV" (*30*)); (2) outer and inner RLOOCV ("Nested RLOOCV"); (3) outer LOOCV with inner 5-fold cross validation ("5-fold LOOCV"); and (4) outer RLOOCV with inner 5-fold cross-validation ("5-fold RLOOCV"). In all cases, the nested cross-validation was used to tune the L^2 regularization of logistic regression models. (**A**) Synthetic simulations on randomly generated data (as in **Figs. 2,3**; **Methods**). (**B**) ROC curve of models trained on the classification task from Lozano et al. (*42*) (as in **Fig. 5C**). (**C**) Results on classification tasks from data published in the UCIMLR, as in **Fig. 4A**, for logistic regression models evaluated either with Honest LOOCV (i.e. nested LOOCV) or nested RLOOCV.

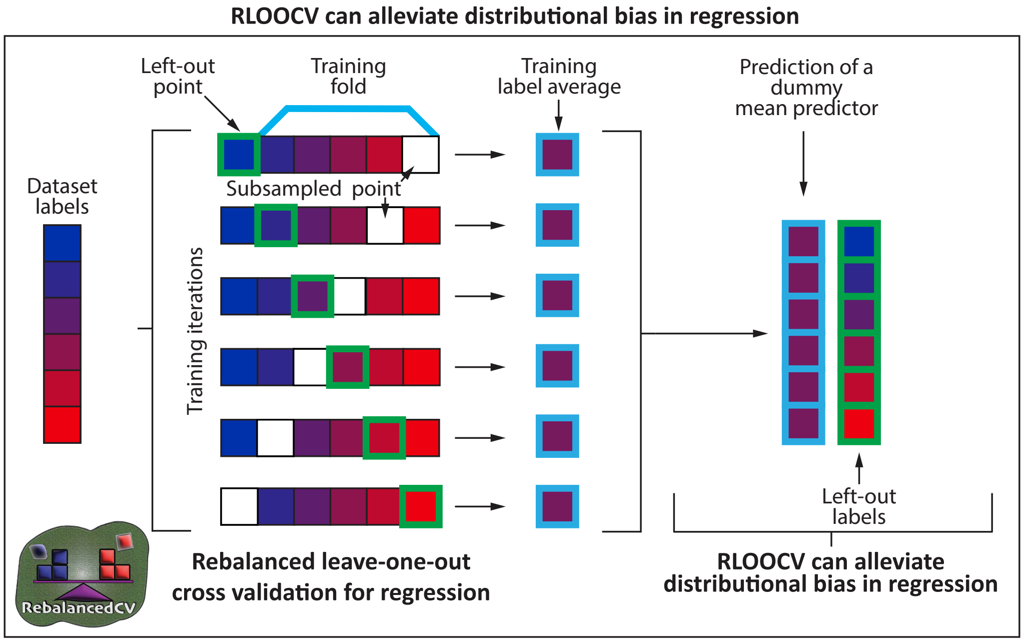

Fig. S7.

RLOOCV can alleviate distributional bias in regression. An illustration of our proposed rebalanced LOOCV (RLOOCV) approach for regression. For each test instance (or fold), we remove from the training set a data instance that is as close to the opposite of the held-out test-set difference to the dataset mean, while not surpassing this value (i.e. the supremum). Doing this alleviates the impact of distributional bias on the dataset without risking an overcorrection that could erroneously inflate the evaluated performance.

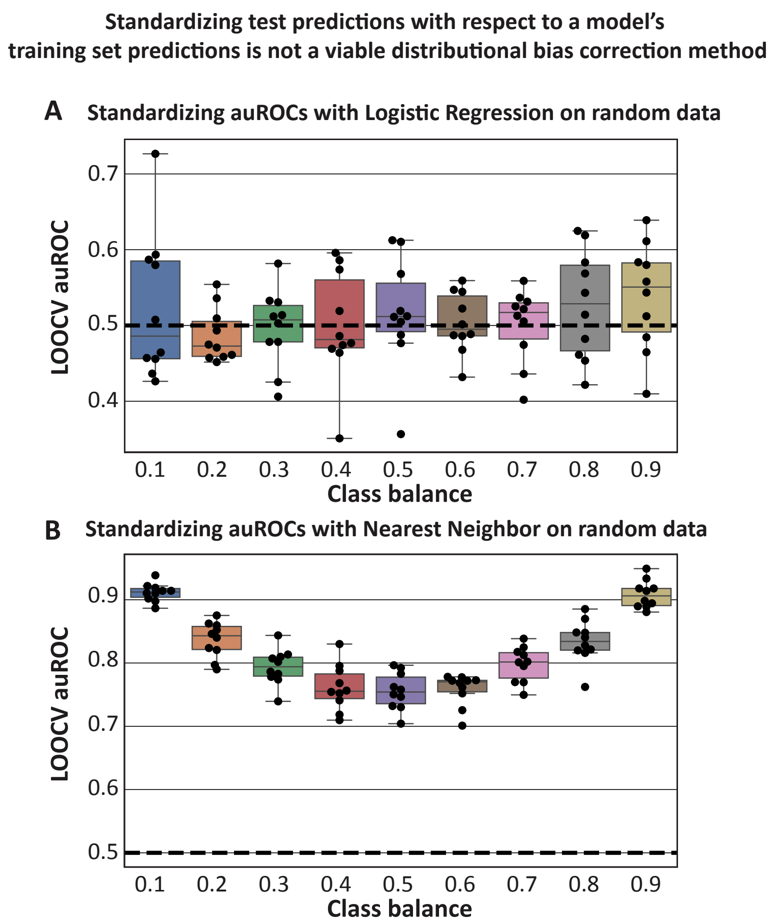

Fig. S8.

Post-hoc prediction standardization may lead to over-evaluation of performance. Boxplots demonstrating a similar analysis as in **Fig. 2A**, but all test predictions are standardized to the model's predictions on the training set via z-scoring. (**A**) Logistic regression evaluation standardized in this way produces results similar to the expected random guess, with an auROC close to 0.5 ($p = 0.14$ via a single one-sample t-test of the aggregated results). (**B**) 1-Nearest Neighbor models in the same standardized evaluation framework yield results larger than 0.5 ($p < 0.001$ via a single one-sample t-test of the aggregated results). Box, IQR; line, median; whiskers, nearest point to 1.5*IQR.

REFERENCES AND NOTES

1. M. S. Boyce, P. R. Vernier, S. E. Nielsen, F. K. A. Schmiegelow, Evaluating resource selection functions. *Ecol. Model.* **157**, 281–300 (2002).

2. Y. Liu, T. Han, S. Ma, J. Zhang, Y. Yang, J. Tian, H. He, A. Li, M. He, Z. Liu, Z. Wu, L. Zhao, D. Zhu, X. Li, N. Qiang, D. Shen, T. Liu, B. Ge, Summary of ChatGPT-Related research and perspective towards the future of large language models. *Meta Radiol.* **1**, 100017 (2023).

3. K. He, X. Zhang, S. Ren, J. Sun, Deep residual learning for image recognition. arXiv:1512.03385 [cs.CV] (2015).

4. O. P. Jena, B. Bhushan, N. Rakesh, P. N. Astya, Y. Farhaoui, *Machine Learning and Deep Learning in Efficacy Improvement of Healthcare Systems* (CRC Press, 2022).

5. Y. Lecun, L. Bottou, Y. Bengio, P. Haffner, Gradient-based learning applied to document recognition. *Proc. IEEE* **86**, 2278–2324 (1998).

6. S. Raschka, Model evaluation, model selection, and algorithm selection in machine learning. arXiv:1811.12808 [cs.LG] (2018).

7. A. J. Thirunavukarasu, D. S. J. Ting, K. Elangovan, L. Gutierrez, T. F. Tan, D. S. W. Ting, Large language models in medicine. *Nat. Med.* **29**, 1930–1940 (2023).

8. S. Whalen, J. Schreiber, W. S. Noble, K. S. Pollard, Navigating the pitfalls of applying machine learning in genomics. *Nat. Rev. Genet.* **23**, 169–181 (2022).

9. K. Giannakopoulos, A. Kavadella, A. Aaqel Salim, V. Stamatopoulos, E. G. Kaklamanos, Evaluation of the performance of generative AI large language models ChatGPT, Google Bard, and Microsoft Bing Chat in supporting evidence-based dentistry: Comparative mixed methods study. *J. Med. Internet Res.* **25**, e51580 (2023).

10. F. Zhang, *Cross-Validation and Regression Analysis in High-Dimensional Sparse Linear Models* (Stanford University, 2011).

11. M. Stone, Cross-validatory choice and assessment of statistical predictions. *J. R. Stat. Soc. B. Methodol.* **36**, 111–133 (1974).

12. S. Arlot, A. Celisse, A survey of cross-validation procedures for model selection. *Statist. Surv.* **4**, 40–79 (2010).

13. Y. Zhang, Y. Yang, Cross-validation for selecting a model selection procedure. *J. Econom.* **187**, 95–112 (2015).

14. T. Fawcett, An introduction to ROC analysis. *Pattern Recognit. Lett.* **27**, 861–874 (2006).

15. D. M. W. Powers, Evaluation: From precision, recall and F-measure to ROC, informedness, markedness and correlation. arXiv:2010.16061 [cs.LG] (2020).

16. S. Varma, R. Simon, Bias in error estimation when using cross-validation for model selection. *BMC Bioinformatics* **7**, 91 (2006).

17. R. J. Tibshirani, R. Tibshirani, A bias correction for the minimum error rate in cross-validation. *Ann. Appl. Stat.* **3**, 822–829 (2009).

18. R. J. Tibshirani, S. Rosset, Excess optimism: How biased is the apparent error of an estimator tuned by SURE? *J. Am. Stat. Assoc.* **114**, 697–712 (2019).

19. S. Bates, T. Hastie, R. Tibshirani, Cross-validation: What does it estimate and how well does it do it? *J. Am. Stat. Assoc.* **119**, 1434–1445 (2024).

20. W. A. Yousef, On the smoothness of cross-validation-based estimators of classifier performance. arXiv:1907.13413 [stat.ML] (2019).

21. B. Wang, A. Yang, A consolidated cross-validation algorithm for support vector machines via data reduction. *Adv. Neural Inf. Process. Syst.* **35**, 394–405 (2022).

22. S. Arlot, M. Lerasle, Choice of V for V-fold cross-validation in least-squares density estimation. *J. Mach. Learn. Res.* **17**, 1–50 (2016).

23. R. Kohavi, “A study of cross-validation and bootstrap for accuracy estimation and model selection” in *IJCAI’95: Proceedings of the 14th International Joint Conference on Artificial Intelligence—Volume 2* (Morgan Kaufmann Publishers Inc., 1995), pp. 1137–1143.

24. R. R. Picard, R. Dennis Cook, Cross-validation of regression models. *J. Am. Stat. Assoc.* **79**, 575–583 (1984).

25. L. Breiman, P. C. Spector, Submodel selection and evaluation in regression. The X-random case. *Int. Stat. Rev.* **60**, 291–319 (1992).

26. S. Geisser, A predictive approach to the random effect model. *Biometrika* **61**, 101–107 (1974).

27. Y. Luo, Z. Ren, R. Barber, “Iterative approximate cross-validation” in *ICML’23: Proceedings of the 40th International Conference on Machine Learning* (PMLR, 2023), pp. 23083–23102.

28. P. C. Bellec, Out-of-sample error estimate for robust M-estimators with convex penalty. arXiv:2008.11840 [math.ST] (2020).

29. B. Avelin, L. Viitasaari, Concentration inequalities for leave-one-out cross validation. arXiv:2211.02478 [math.ST] (2022).

30. B. Wang, H. Zou, Honest leave-one-out cross-validation for estimating post-tuning generalization error. *Stat* **10**, e413 (2021).

31. A. Luntz, On estimation of characters obtained in statistical procedure of recognition. *Techn. Kibern.* (1969).

32. A. Celisse, Optimal cross-validation in density estimation with the L^2-loss. *Ann. Statist.* **42**, 1879–1910 (2014).

33. M. Pahar, M. Klopper, R. Warren, T. Niesler, COVID-19 cough classification using machine learning and global smartphone recordings. *Comput. Biol. Med.* **135**, 104572 (2021).

34. D. B. Suits, Use of dummy variables in regression equations. *J. Am. Stat. Assoc.* **52**, 548–551 (2012).

35. Z. Zhong, L. Zheng, G. Kang, S. Li, Y. Yang, Random erasing data augmentation. *AAAI* **34**, 13001–13008 (2020).

36. M. A. Tanner, W. H. Wong, The calculation of posterior distributions by data augmentation. *J. Am. Stat. Assoc.* **82**, 528–540 (1987).

37. A. Mumuni, F. Mumuni, Data augmentation: A comprehensive survey of modern approaches. *Array* **16**, 100258 (2022).

38. G. I. Austin, A. Brown Kav, S. ElNaggar, H. Park, J. Biermann, A.-C. Uhlemann, I. Pe'er, T. Korem, Processing-bias correction with DEBIAS-M improves cross-study generalization of microbiome-based prediction models. *Nat. Microbiol.* **10**, 897–911 (2025).

39. UCI Machine Learning Repository. https://archive.ics.uci.edu/.

40. J. M. Fettweis, M. G. Serrano, J. P. Brooks, D. J. Edwards, P. H. Girerd, H. I. Parikh, B. Huang, T. J. Arodz, L. Edupuganti, A. L. Glascock, J. Xu, N. R. Jimenez, S. C. Vivadelli, S. S. Fong, N. U. Sheth, S. Jean, V. Lee, Y. A. Bokhari, A. M. Lara, S. D. Mistry, R. A. Duckworth 3rd, S. P. Bradley, V. N. Koparde, X. V. Orenda, S. H. Milton, S. K. Rozycki, A. V. Matveyev, M. L. Wright, S. V. Huzurbazar, E. M. Jackson, E. Smirnova, J. Korlach, Y.-C. Tsai, M. R. Dickinson, J. L. Brooks, J. I. Drake, D. O. Chaffin, A. L. Sexton, M. G. Gravett, C. E. Rubens, N. R. Wijesooriya, K. D. Hendricks-Muñoz, K. K. Jefferson, J. F. Strauss III, G. A. Buck, The vaginal microbiome and preterm birth. *Nat. Med.* **25**, 1012–1021 (2019).

41. C. Huang, C. Gin, J. Fettweis, B. Foxman, B. Gelaye, D. A. MacIntyre, A. Subramaniam, W. Fraser, N. Tabatabaei, B. Callahan, Meta-analysis reveals the vaginal microbiome is a better predictor of earlier than later preterm birth. *BMC Biol.* **21**, 199 (2023).

42. A. X. Lozano, A. A. Chaudhuri, A. Nene, A. Bacchiocchi, N. Earland, M. D. Vesely, A. Usmani, B. E. Turner, C. B. Steen, B. A. Luca, T. Badri, G. S. Gulati, M. R. Vahid, F. Khameneh, P. K. Harris, D. Y. Chen, K. Dhodapkar, M. Sznol, R. Halaban, A. M. Newman, T cell characteristics associated with toxicity to immune checkpoint blockade in patients with melanoma. *Nat. Med.* **28**, 353–362 (2022).

43. T. Chen, C. Guestrin, “XGBoost: A scalable tree boosting system” in *Proceedings of the 22nd ACM SIGKDD International Conference on Knowledge Discovery and Data Mining* (Association for Computing Machinery, 2016), pp. 785–794.

44. J. H. Friedman, Greedy function approximation: A gradient boosting machine. *Ann. Stat.* **29**, 1189–1536 (2001).

45. T. Vogl, I. N. Kalka, S. Klompus, S. Leviatan, A. Weinberger, E. Segal, Systemic antibody responses against human microbiota flagellins are overrepresented in chronic fatigue syndrome patients. *Sci. Adv.* **8**, eabq2422 (2022).

46. X. Sun, W. Xu, Fast implementation of DeLong’s algorithm for comparing the areas under correlated receiver operating characteristic curves. *IEEE Signal Process. Lett.* **21**, 1389–1393 (2014).

47. L. Breiman, Bagging predictors. *Mach. Learn.* **24**, 123–140 (1996).

48. G. I. Austin, T. Korem, Planning and analyzing a low-biomass microbiome study: A data analysis perspective. *J. Infect. Dis.*, **jiae378** (2024).
